# Examining Management and Employees’ Perceptions of Occupational Heat Exposure and the Effectiveness of a Heat Stress Prevention Intervention on Safety and Well-Being among Natural Gas Construction Workers: A Qualitative Field-Based Study

**DOI:** 10.3390/ijerph21091255

**Published:** 2024-09-21

**Authors:** Muinat Abolore Idris, Christine Markham, Kristina D. Mena, William B. Perkison

**Affiliations:** 1 Department of Environmental and Occupational Health Sciences, University of Texas Health Science Center at Houston School of Public Health, Houston, TX 77030, USA; kristina.d.mena@uth.tmc.edu (K.D.M.); william.b.perkison@uth.tmc.edu (W.B.P.); 2 Department of Human Promotion and Behavioral Sciences, University of Texas Health Science Center at Houston School of Public Health, Houston, TX 77030, USA; christine.markham@uth.tmc.edu

**Keywords:** heat stress, construction workers, heat stress prevention program, heat-related conditions, worker safety, well-being, facilitators, barriers

## Abstract

Background: Numerous risk factors have been identified as significantly influencing outdoor workers’ risk for heat stress and heat-related conditions, impacting their health, well-being, and productivity. However, the specific effects of these factors on construction workers’ safety, health, and well-being remain under-researched. With climate change increasing temperatures, assessing heat stress among construction workers is imperative. Objective: To identify the barriers and facilitators influencing the safety of natural gas construction workers and evaluate an implemented heat stress intervention. Methods: In the summer of 2023, two semi-structured interviews and six focus groups were conducted with twenty-one stakeholders at a Texas natural gas construction site. Results: Key facilitators include employee preparedness, use of employer-provided resources, hydration logs, and real-time communication tools. Contrarily, the barriers include daily work schedules, access to dehydrating beverages, and generational differences with the non-implementation of mandatory rest breaks. The heat stress program was perceived as effective, surpassing recommended guidelines. Conclusion: To advance construction workers’ safety, health, and well-being, both employee involvement and employer management are needed, along with no-cost accessible resources. Additionally, implementing a required routine rest break and comprehensive heat stress education, particularly for older workers, will significantly promote safety and safe work practices in hot environments. Note: in this study, the terms ‘worker’ and ‘employee’ are used interchangeably.

## 1. Introduction

In both the USA as well as worldwide, construction workers represent one of the occupational groups most vulnerable to heat stress and its associated health outcomes. They account for at least one of three work-related heat deaths [1] and are at 13 times higher risk of heat-related fatality compared to workers in general industries within the USA [2]. Between 2011 and 2022, the Bureau of Labor Statistics (BLS) Census of Fatal Occupational Injuries reported 479 heat-related fatalities, with 141 occurring within the construction industry [3]. This elevated risk is directly linked to excessive occupational heat exposure, a well-established contributing factor to heightened heat-related conditions among construction workers. Gariazzo et al. (2023) [4] found that exposure to high temperatures was associated with more severe injuries among Italian construction workers between 2014 and 2019, with a relative risk (RR) of 1.361 (95% CI: 1.068–1.733). Similarly, Calkin et al.’s study conducted in Washington State, USA, reported a positive association between occupational heat exposure and the risk of traumatic injuries among outdoor construction workers [5].

Various factors contribute to the increased heat-related injury risk among workers, including personal attributes [6], workplace conditions such as high ambient temperatures, metabolic heat, physical discomforts [5,7], fogged-up safety glasses, accidental contact with hot surfaces [8], and lack of experience [9]. Additionally, heat stress symptoms, including impaired cognitive function, dizziness, slowed response time, muscle fatigue and cramping [5,7], and loss of concentration and coordination [8] may further elevate workers’ risk of injury in hot environments. The impact of these factors on construction workers’ safety, health, and well-being remains unclear, highlighting the urgent need for further investigation, particularly in the context of climate change and rising temperatures.

These heat stress risks to workers continue to increase due to rising global temperatures. In the USA, climate projections indicate a decrease in the number of freezing days and an increase in days with temperatures surpassing 90 °F (32.2 °C) with an expected rise of 2.5 °F (1.4 °C) between 2021 and 2050 compared to the preceding years (1976–2005) [10]. States like Arizona, Nevada, California, Texas, and Florida may experience more negative effects from the rising temperatures. For instance, from 2016 to 2020, heat-related deaths reported during the summer months ranged from 10 to 1170, with Arizona reporting the highest number (*n* = 1170), followed by Nevada (*n* = 558), California (*n* = 448), and Texas (*n* = 384) [11]. Given this changing heat paradigm, assessing and addressing heat stress-related risks among workers is critically important, along with identifying effective mitigation strategies.

We aimed to identify the barriers and facilitators influencing the safety of natural gas construction workers who build oil and gas refineries, specifically in relation to heat stress. We also evaluated the perceived effectiveness of an implemented heat stress intervention. These workers are classified under the National American Industry Classification System (NAICS) 238,990—“All Other Specialty Trade Contractors” category. Our decision to specify the NAICS code reflects the recognition that injury and illness risks may vary across different construction sub-sectors. For instance, a study evaluating the Washington State Fund workers’ compensation system from 2000 to 2012 found that of the 63,720 compensation claims, the majority were from the NAICS 238,000 “specialty trades contractors” (67.1%), followed by NAICS 236,100 “construction of buildings-residential” (18.1%) [5].

In our study, three research questions were addressed: (i) What are the barriers to heat stress prevention affecting natural gas construction workers’ safety? (ii) What are the facilitators to heat stress prevention affecting this population? (iii) How does the implementation of a heat stress intervention influence workers’ safety in relation to heat stress?

## 2. Materials and Methods

### 2.1. Study Design

Due to the site’s workload and number of participants recruited, two in-depth semi-structured key informant interviews and six focus groups were conducted to (i) explore the barriers and facilitators influencing workers’ safety in a hot environment; and (ii) understand participants’ knowledge of heat-related injuries (HRIs) and the available heat stress prevention program.

### 2.2. Study Site and Location

This study was conducted at one location (Houston, TX, USA) of a natural gas construction company that has 21 locations across the USA and one in India. This company integrates engineering, procurement, and construction services in solving engineering and construction challenges, particularly in the petrochemical, energy, and heavy industrial sectors. The site operates two work shifts (morning: 8:00 a.m.–4:30 p.m.; evening: 5:00 p.m.–1:30 a.m.). During the Summer of 2023, the company adjusted the morning shift work hours to 5:00 a.m.–3:00 p.m. to reduce workers’ daily heat exposure.

### 2.3. Study Population and Participant Recruitment

Key informant stakeholders, including safety professionals and construction workers, were recruited. The recruitment was aided by a priori knowledge and recommendations from the research team and company associates. A one-page flyer was provided to our company’s contact person to introduce the study before screening and recruiting participants. A recruitment flyer and a screener form were designed. Before posting the recruitment flyer, the researcher spoke briefly about the study to the employees during one of their general Wednesday weekly morning meetings. After the general meeting, the research team briefly met with the safety professionals to discuss the study in detail, and then sent an email inviting the safety professionals to participate in the study. The recruitment flyer was posted on the work site at the break room and other strategic locations. A translated version (Spanish) of the flyer was also posted at the work site.

On each study day, participants were screened for eligibility via a screener form and recruited based on purposive sampling. Inclusion criteria included daytime work schedules and being over 18 years of age. Workers who worked indoor shifts during the study period were excluded. Each focus group included two to five participants.

### 2.4. Study Materials

A study protocol, semi-structured interview, and focus group guide (see Supplementary Materials, Interview and focus group guide main questions) were developed to help structure the discussions around the research questions. These guides included open-ended questions and were pilot-tested for validity. The focus group guide was translated into a Spanish version.

### 2.5. Data Collection Procedures

A risk management plan was developed before collecting the data in the field. This helped us identify, assess, and mitigate potential risks, including time delays, changes in participants’ work schedules, and weather disruptions that might arise during the study. Interviews and focus groups were moderated by proficient researchers (PhD candidate and Graduate Teaching Assistant), with detailed field notes taken by a notetaker (PhD student) experienced in qualitative research to collect information that included participants’ casual and structured observations, verbatim quotes, and paraphrased responses. Oral informed consent was obtained from participants, confidentiality was ensured, and each participant was provided with a copy of the consent form. Participation was voluntary. Data collection occurred in August 2023, with each interview lasting 15–40 min and each focus group lasting approximately 1–1:20 h. Participants were provided lunch and a $20.00 gift card as an incentive. Data were collected using field notes and audio tape recording. All discussions were audio recorded and transcribed verbatim for analysis.

### 2.6. Data Analysis

Electronic data were imported into ATLAS/ti 23 (ATLAS.ti scientific software development GmbH, Technical University, Berlin, Germany). The data were formatted with consistent speaker identifiers (speakers ID), with line breaks between speakers. Data were analyzed using the Framework Analysis developed by the National Center for Social Research to generate policy and practice-orientated findings explicitly [12]. Firstly, we familiarized ourselves with the data to preserve participants’ narratives by listening to recording tapes and re-reading field notes and transcript data until fully immersed. Using an inductive coding approach and thematic analysis, we developed a coding scheme (codebook) based on quotes extracted from the data. Codes were assigned to describe each content and then sorted into potential themes by identifying patterns in the codes across transcripts. Codes were translated systematically across all data sets, and similar codes were grouped into subthemes, which were further consolidated into broader emerged themes. The themes were defined, named, and written up. To minimize bias, the research team reviewed and validated the developed codes and themes. Data mapping summaries and word clouds were generated to interpret data and compare patterns for visual representation. To increase the credibility of our findings, the analysis’s reliability and validity, quotes from transcripts were used as examples of specific definitions.

## 3. Results

### 3.1. Participants

Data from two safety officers and 19 workers were analyzed (Table 1). The focus groups included participants with diverse job titles: electrician (*n* = 2), instrument technology helper (*n* = 1), pipe fitter helper (*n* = 1), paint helper (*n* = 2), painter (*n* = 10), scaffold carpenter (*n* = 1), pipe fitter (*n* = 1), and concrete finisher (*n* = 1). The electricians typically spend between 20 and 30% of their time working inside and 70–80% outside, but worked exclusively outside during the data collection.

### 3.2. Content Findings

**Figure 1:**
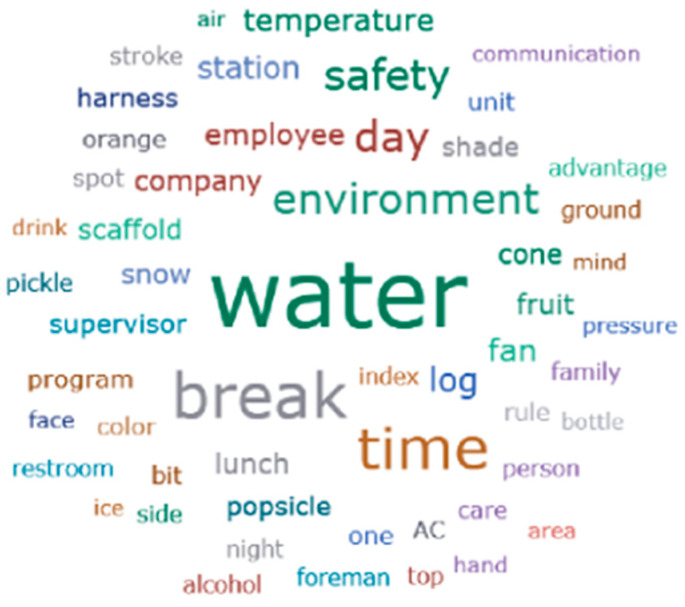
Visual interpretation of participants’ keywords mentioned in relation to their working conditions.

Figure 1 illustrates the most frequently mentioned words from both focus groups and key informant semi-structured interviews. The predominant words were ‘water (*n* = 263)’ and ‘break (*n* = 166)’ (Table 2).

### 3.3. Overview of Findings

**Figure 2:**
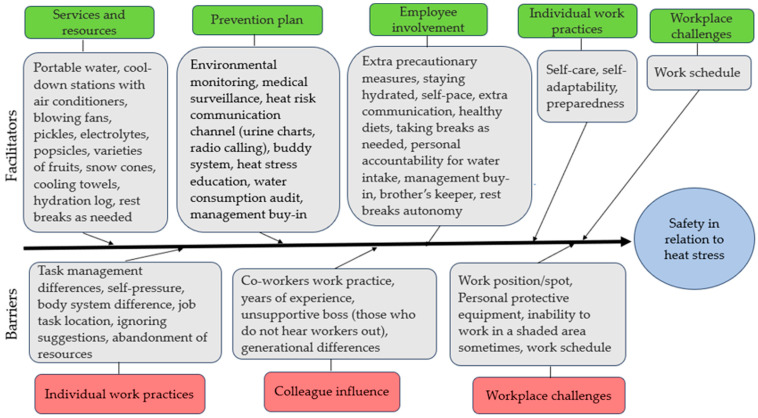
Barriers and facilitators that affect worker safety.

Using a fishbone diagram adapted from a qualitative study conducted by Al-Janabi et al. (2019) [13], Figure 2 summarizes the identified facilitators and barriers. Three themes and eight subthemes emerged from participants’ knowledge, experiences, feelings, and opinions (Table 3), describing the barriers, facilitators, and participants’ perceived effectiveness of the implemented heat stress prevention program.

#### 3.3.1. Heat Stress Intervention Context

This theme delineates key components of the heat stress prevention program, focusing on safety, mitigating heat-related conditions, and enhancing workers’ overall health and well-being. It describes the written program’s policy, which was last reviewed in November 2022, and also explores facilitators as well as evaluates the program’s perceived effectiveness. The program is mainly designed and disseminated during the Summer (May–September) period.

*A.*  *Services and resources to prevent heat-related conditions*

All the participants described the employer-provided resources as helpful safety facilitators. These resources are provided at no cost to all employees, including subcontractors. The resources include portable water stored in ice chests/coolers stationed across the job sites, blowing fans strategically placed across the job sites, air-conditioned cool down stations, electrolyte drinks, water totes filled with pickles, a variety of fruits supplied on weekdays, and once-weekly snow cones from a local mobile snow cone truck vendor (Figure 2). These resources enhance hydration with lesser cramps and foster a refreshed sensation among workers. Employees were also provided cooling towels to suppress their core body temperature (CBT). Participants reported that these towels aid in quicker body temperature regulation. Employees are granted 10–15 min breaks, with flexibility for longer breaks as needed. Break-taking was not mandatory.

**Figure 3:**
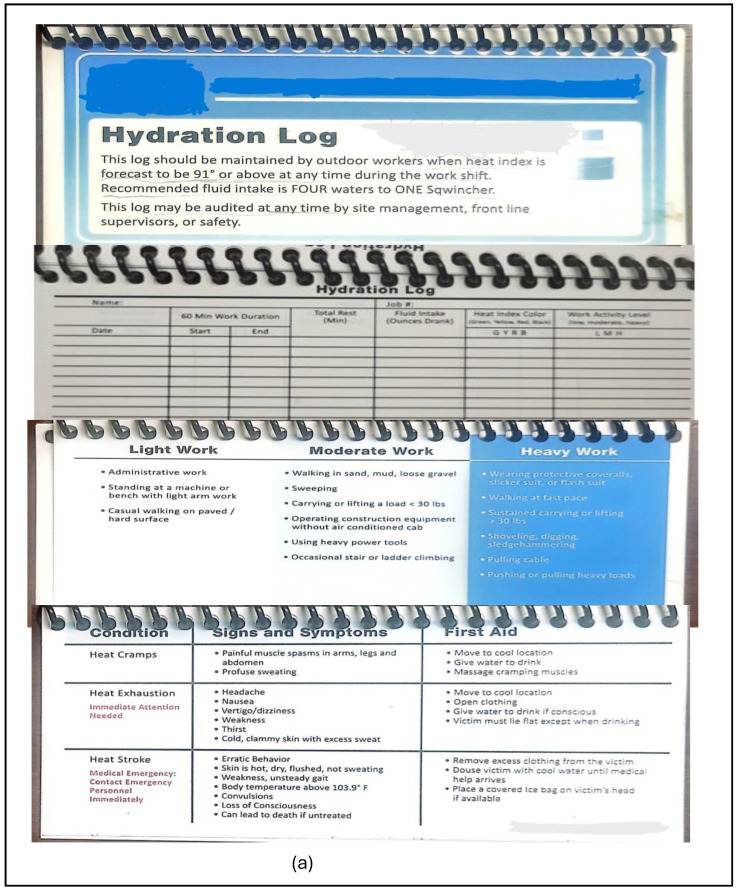

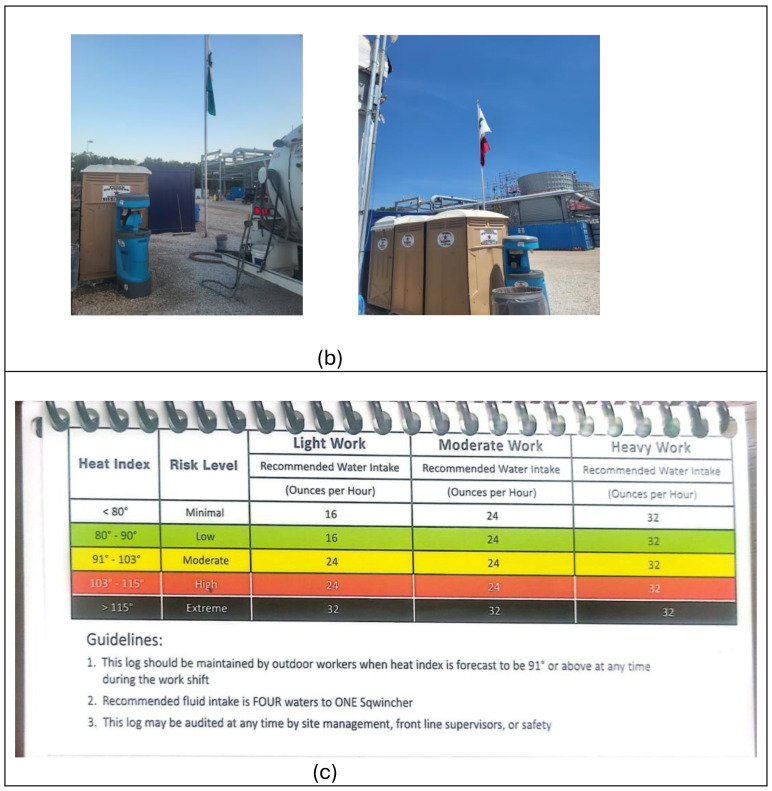

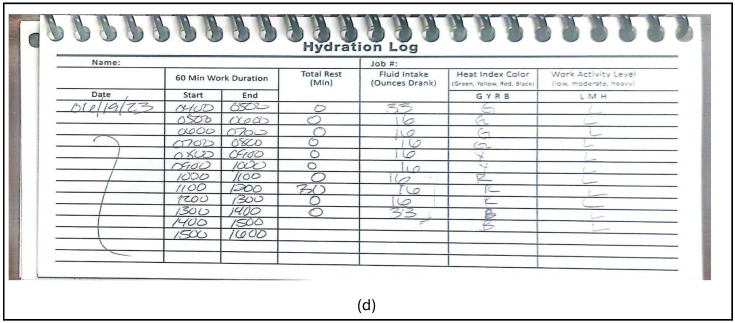
(**a**) Waterlog/hydration log content; (**b**) visual on-site heat index tool (heat index flag); (**c**) individual accessible heat index chart from the waterlog; (**d**) sample of a filled waterlog.

To continue promoting safety, the employer provides a small booklet called a waterlog/hydration log (Figure 3a), which all employees must complete periodically. This booklet educates employees on the recommended water intake per the on-site heat index levels and workload expectations, and serves as an assessment tool to identify factors contributing to any heat-related conditions experienced during job tasks. Participants reported using the waterlog to gauge their water consumption, referencing that it helps them regulate their water consumption in accordance with recommended levels. Additionally, it provides guidance on recommended water-to-electrolyte ratios and offers strategies to mitigate heat stress symptoms if encountered (Figure 3a). This participant describes the waterlog purpose and use in detail.

*“… just in case like something were to happen, if you were to get hurt or heat stress, the first thing they’re [employer/safety officer] going to look at is this [waterlog]… They’re going to say, hey, …, has he been drinking sufficient water? Yes, around 6:30 he has ounces, 32, 16, whatever it is. Has he been keeping up with him? And yes he’s been keeping up with it to stay safe and take care of it [heat exposure]”*

[Painter ID: 4].

*B.*  *Heat stress prevention plan and worker safety*

The employer has a written heat stress prevention policy in place, which includes daily monitoring and auditing of employees’ water consumption by supervisors/foremen and safety officers to ensure employees stay hydrated throughout the day. As written in the policy that our contact person (site manager) shared with us, the policy was last reviewed in November 2022.

Participants identified the buddy system, environmental monitoring, and safety precautions as some facilitators of workers’ safety. These precautions include utilizing heat stress communication tools like urine color charts. Educating workers on heat stress and personal lifestyle factors, including sleeping patterns, during toolbox talks and weekly safety meetings, and using occupational heat stress-related video clips as another educational tool. Participants described the heat index level flags (Figure 3b) and a displayed digital heat index level readout as a visible communication channel to all employees throughout the work shift. These tell all employees their heat exposure temperature range, heat index level, heat risk level, type of work they should engage in at certain times, and the daily projected heat index level. Additionally, each employee has access to a copy of the heat index level chart, which can be found in the waterlog, which reminds employees of their heat stress risk levels and the amount of water to drink per hour (Figure 3c) to stay hydrated and maintain safety. A safety officer describes these tools and the safety measures all employees should follow.

*“We have urine color charts all over the place inside the port-a-can so the guys understand, hey, I’m peeing dark orange, that means I’m very dehydrated, versus I’m peeing clear, because I’ve had enough water …, they’re hearing all calls, explaining over the radio, the temperatures, and they got the flags flying, green, yellow, red, or black [depending on the temperature and the heat index levels]. … they see when they walk down the unit, they see two flags and it tells them, is it green, yellow, or red? Saying no, is it hot? There’s a big digital readout right there. And that scrolls showing the temperature right now, the projected time we’re going to go into the next category, on to the end of the day. … We’re making all calls throughout the day to make them aware that, hey, it’s 115 degrees. Work in the shade, take breaks, drink water”.*

[Safety officer ID: 21].

*C.*  *Employee involvement and safety measures*

Employees’ involvement was the primary facilitating factor, and participants described the following as some of the facilitators: compliance with the heat stress prevention program; utilizing the employer-provided resources; supporting each other (brothers’ keeper, i.e., looking out for one another); stakeholder’s decision-making participation; and autonomy to taking breaks (Figure 2). An electrician (ID: 13) described his experience regarding participation in stakeholder decision-making:* “they [employer] tell us, hey, what else y’all think we could use? Like, I know, last summer [2022] somebody mentioned snow cones. And they started bringing snow cones every Friday”.* Another participant describes personal accountability for water intake using the waterlog as a facilitator. Figure 3d is an example of a filled-out waterlog.

*“And on there [waterlog] you put out the hourly, … you write the date down and the time. You can put one hour, 17 o’clock, how many ounces of water did you drink? It could be 16, 32. If you had more, put that down … When … the temperature hits like a 100 or 100 plus, they want you to keep taking breaks every other hour, …”*

[Painter ID: 6].

Influencing healthy diets was another safety measure in place to facilitate workers’ safety. One of the safety officers (ID: 20) described this as one of the management strategies by discouraging the consumption of carbonated, energy, and alcoholic beverages and not allowing such drinks on-site because they dehydrate.

All the focus group participants described carbonated beverages, including soda, as a safety barrier because they are dehydrating agents.

*“… when you [employees] come in the morning, … when you clock in, there’s supervisors waiting at the gate, checking everyone’s box, looking for energy drinks or anything that can dehydrate you and as well as the vendors we already on an agreement we already told them that they can’t sell any energy drinks period on this job”.*

[Safety officers ID: 20].

#### 3.3.2. Factors Influencing Workers’ Safety

This theme summarizes additional facilitators and all the barriers influencing workers’ safety.

*A.*  *Individual work practices and self-care*

Participants identified factors, including individual task management differences, job location (working in an elevated area versus ground level), preparedness, abandoning or abusive use of employer-provided resources (like not drinking water, overconsumption of water or electrolytes without water) as either facilitators or barriers to safety. *“overdoing Squincher [electrolyte solution drink] is dangerous *(Helper painter ID: 9); *it puts employees at risk of not sweating *(Painter ID: 10); *and getting nauseous if you [employee] drink 8 to 10 of it [electrolyte beverage]”* (Painter ID: 12).

Participants described preparing themself the night before, avoiding heavy dinners, eating light lunches [“*heavy lunch makes you kind of tired, drowsy*. (Painter ID: 17)”], consuming vegetables, steering clear of carbonated drinks and alcohol [“*That soda, it drains you* (Painter ID: 17)”] as individual key factors facilitating employee safety. A helper painter (ID: 18) mentioned that she tries not to drink during the week to avoid being hungover because alcohol dehydrates and being dehydrated is unsafe (pipe fitter helper ID: 2). Contrary to the help painter’s (ID: 18) opinion, a participant (Painter ID: 15) mentioned that drinking “a beer or two is fine but alcohol, hard liquor are not good.” Furthermore, this participant (Painter ID: 15) emphasized the importance of preparing the night before as a crucial facilitator to safety and health.

*“… Everything that you want to do in that sun, it starts the day before. You want to make sure you get good sleep, you want to eat good, you want to make sure you get plenty of water, make sure you’re putting the salt in your body, so that’ll carry you on to the next thing [day]. Drink plenty of water. The day before, Yeah, so it will be in you before the next day. … You don’t want to start today and start drinking water. It will be too late. … So by the time you start sweating, you got water in you. Instead of you sweating now and trying to, I’m trying to put water back in you. To stay hydrated. …”*

[Painter ID: 15].

Additionally, participants described self-care as a safety facilitator and barrier. Participants mentioned that as construction workers, working in the heat is inevitable; employees can increase their resistance by being aware of their body’s needs and adjusting their work based on how they feel because only the employee technically knows their body (Painter ID: 10). This self-care type can help facilitate safety.

*“… You get paid by the hour. You don’t have to rush nothing. Take your time. You don’t have to move with nobody. Move at your own pace. You know your body. It’s not a car racing out here”.*

[Painter ID: 10].

Contrary to this participant’s (Painter ID: 10) self-care description, working under self-pressure hinders employees’ safety. This participant described how his self-pressure task management could hinder his safety.

*“… sometimes the task can be like something very tedious so you have to sit there with it patiently while the Sun’s beating down on you, you know … you have to really key in on what you’re doing because if not you’re messing up and so just all that pressure … from trying to make sure that this gets done properly and having the Sun beat down on you …, definitely affects you negatively”*

[Concrete finisher ID: 3].

*B.*  *Colleagues’ influence on work practices (co-worker and supervisor)*

Participants described co-workers’ work practices, generational differences, etc., as barriers to employees’ safety. Participants mentioned that workers in the industry in the past two to three decades often work the same way they used to without taking breaks. The older generations rarely take breaks, working from morning until lunchtime without rest, despite the changing weather and climate conditions. This employee shared her experience with the older generations.

*“…, the old school, they don’t want to take a break. … It’s more [not taking break] of a right thing to them. …”*

[Helper painter ID: 18].

This safety officer also shared how co-workers’ work practices hinder employees’ safety.

*“Sometimes people will allow themselves to get pushed past their limit because of that team environment…My personal experiences is when you’re working in a crew, that’s basically a team. Say you’re a scaffold builder and you got eight guys in your crew, or a wire puller you got 20 guys in your crew If one guy stops, the whole operation stops. That creates pressure you know that’s a stressor. … But if that guy (doing concrete work) that’s trying to quit, he wants to quit and everybody else had to quit too, well that would make him think, maybe I can push a little bit more. Maybe I’ll wait for the next guy. And maybe the next guy is thinking the same thing, I will be waiting for the next guy. Once the next guy is thinking the same thing, … before you know it, you’ve got a whole group of guys that are very close to overheating”.*

[Safety officer ID: 21].

*C.*  *Workplace challenges*

Employees identified their work position/spot, which determines their work posture, working in an elevation zone, PPE including harnesses, spoggles, dust masks, etc., as safety barriers. Participants mentioned that working in the heat makes (i) the spoggles fogged up because it is hot and humid [Helper painter ID: 18] and (ii) the dust mask suffocates [Painter ID: 12]. This employee described how his work position can influence his safety.

*“… like right now I’m in a tight spot, … And everything ain’t in the good spot. I can’t stand up straight, If I’m in an open spot it’s all good but if I’m in a tight spot no I cannot move like I want to move. I gotta move with it, not against it. … Basically I gotta stay more focused in a tight spot. Because I can just turn around and bum hit my head. … I’m tall, they put me in the spot where she [Helper painter ID: 9] should be at. And it’s not comfortable”.*

[Painter ID: 10].

All participants described working on the scaffold as a critical safety barrier. This employee clarifies how working at an elevated work zone hinders her and other employees from drinking enough water and taking breaks as needed.

*“… Somebody like me whenever I drink water like I have to go to the bathroom, like 5–10 min later. … Not everybody’s the same so it [working on the scaffold] does keep me from drinking as much water because I don’t want to be coming up and down. So I would say that’s frustrating [working on the scaffold], … you might not be able to you know to take that break. You know that you can do [take breaks] if you were on the ground maybe 80% of the time. … Because not all the times you want to keep coming up and down … You’ll burn yourself up before you even get done with your job or even start your job, … then a lot of other guys are like that too especially with the scaffold. … we’ll sit there where we’re at and be like hey pass the water up here you know just to avoid from coming up and down you got a harness you got tools on …”*

[Scaffold Carpenter ID: 7].

Participants also described planning their work in the morning, completing job tasks early to avoid direct sunlight, and working in shaded areas during the peak sun hours (10:30 a.m.–11:45 a.m.) as ways their work schedule facilitates safety. Furthermore, participants shared their perceptions on how different work schedules (full-time employees: 5:00 a.m.–3:00 p.m.; subcontractors: 7:00 a.m.–5:30 p.m.) and working days (13 days on and one day off) facilitate employees’ safety. *“I get off by 3 o’clock that’s when the sun is the hottest, they’ve [employer] done real good, at least on this site”* (Electrician ID: 13). In contrast, some participants described how the schedule and work hours (10 h shifts) could be a potential barrier to their safety. *“We [subcontractors] leave at 5:30 p.m. The hardest time of the day is around 1 [p.m.] to 5 [p.m.]. It gets really hot around 3:00 p.m.”* [Helper painter ID: 18].

#### 3.3.3. Intervention Outcome and Worker Well-Being

This theme outlines participants’ additional perceptions of the implemented heat stress prevention program’s effectiveness. They generally perceived the program as effective in mitigating heat stress, improving safety, and advancing workers’ health and well-being.

*A.*  *Intervention influence on worker well-being and QOL*

Both the safety officers and workers shared similar perceptions about the program. This participant had a positive perception.

*“So, since they [employer-provided resources] are there for the positive effect, I believe that the only way that it will hinder your work is if you ignore the things they’re giving. … It [too much electrolyte] could [cause] stomachaches, cramps, It can put you in a position where you don’t sweat but you‘re taking more of it. …”*

[Painter ID: 11].

Participants also described the employer-provided resources as luxury and a true blessing because some construction sites do not have such opportunities, while some only provide water to employees. This safety officer perceived the program as a massive help to the employees.

*“If we didn’t have any controls or mitigations for these employees and they were just out here working their butts off all day long and we allowed the supervisors to push them [employees], that would be a very bad environment for them. So I think we definitely help them in all aspects, mentally, physically, their well-being at home and their families, all of it. How would I say that? Because if they came out here and just got worked like crazy all day long, they wouldn’t be able to function when they got home. They’d just go home and crash. That’d be the end of them”.*

[Safety officer ID: 21].

## 4. Discussion

This qualitative study explores the factors that facilitate and hinder construction workers’ safety in hot environments and the perceived effectiveness of an implemented heat stress prevention program at a construction site. Providing resources at no cost with easy accessibility, rest break autonomy, and employee preparedness the night before work were key facilitating factors identified at this construction site. These resources, including water and electrolyte supplements, help workers stay hydrated throughout the work shift and perform their job tasks safely. These findings align with previous studies among agricultural workers, which reported that employer provision of clean drinking water and scheduled breaks in the shade, with increased water access, mitigate heat stress impact on kidney function and injury prevention [14,15,16,17,18,19,20]. Ensuring such resources are readily available fosters a supportive environment where workers can take breaks without fear of repercussions, promoting safety and preventing heat stress. Studies show that 95.7% of workers whose employers permit them to take breaks feel comfortable taking breaks to drink water [21], and workers whose employers provided fluids were more satisfied with their breaks compared to workers whose employers do not offer fluids (PR = 1.68; 95% CI = 1.18, 2.39) [14].

Although no federal heat stress standard exists, the OSHA’s “Water. Rest. Shade. (WRS)” initiative emphasizes the importance of hydration and requires employers to provide cool water and cool locations, including shaded areas where workers can take breaks and recover from the heat [22]. Previous research has shown that drinking more water is a behavioral adaptation strategy to prevent heat-related injuries among workers [14,17,18,23,24], particularly in agriculture. Similarly, a study among Chinese construction workers reported that providing cool drinking water as the most common preventive measure workers adopted during hot weather [25]. Additionally, participants in this study described the availability of electrolyte solutions and potassium-rich fruits alongside pickles as helpful resources that further facilitate workers’ safety and increased productivity by restoring workers’ bodies lost essential minerals, fostering hydration, and lessening cramps. These findings are similar to Butler-Dawson et al. (2019) [16], who found an association between increased access to electrolyte solutions and higher electrolyte solutions intake and reduced odds of acute kidney injury among sugarcane workers (OR: 0.94; 95% CI: 0.89–0.99). Similarly, research on Nicaraguan sugarcane workers consistently suggests that consuming electrolyte solutions may lower biomarkers of kidney injury among individuals in high-risk jobs [26]. These indicate that electrolyte supplement provisions not only facilitate safety, but also help to reduce heat-related conditions risk.

This study also suggests that taking breaks, especially in shaded areas with fans or air-conditioners, aids in quick recovery from heat and enhances workers’ safety. Our finding is supported by an agricultural workers’ qualitative study that reported that workers not cooling off in the shade could get dizzy from sweating too much [27]. Although, taking breaks was not scheduled on the study worksite except for a 45 min lunch break, workers could take 10–15 min rest breaks or more to cool down. This 10–15 min break duration is consistent with findings in Saudi Arabian construction [28] and agricultural workers [16,19,20,29].

Additionally, both the workers and safety officers believed that everything related to working safely in a hot environment began the night prior, making preparedness the night before a critical emerging facilitator for safety. This perception aligns with Luque et al. (2019) [29], who reported that excessive alcohol consumption the previous evening contributes to dehydration in agricultural workers. The consumption of soda, energy drinks, beer, and alcohol were identified as dehydrating agents and safety barriers due to the caffeine content. The study site prohibits such drinks, as the employer considers that energy drinks dehydrate. Participants’ responses in this study align with those of Messeri et al. (2019) [30], who reported that their participants perceived that alcoholic beverages aggravate dehydration. However, contrary to our findings and Messeri et al. [30], Luque et al.’s [29] participants did not view beer and alcohol consumption as a barrier; they believed that having a beer at work could quench thirst when water alone was not sufficient. This discrepancy may be due to agricultural employers encouraging the consumption of such drinks. This difference between construction and agricultural employer perception could be a significant contributing factor to the higher risk of heat-related death among agricultural workers.

This study is the first to identify various communication channels, including a hydration monitor tool (waterlog) and emphasized urine color chart, as safety facilitators among this site’s construction workers. These tools help workers recognize dehydration and maintain adequate hydration levels, which is crucial for safety. Similarly, the U.S. Army Public Health Command [31] reported that a urine color test/chart is a simple test to help protect against dehydration, and El Shafei et al. (2018) [32] utilized urine color charts as a hydration status indicator. These findings are significant as they emphasize the role of safe work practices not only in reducing heat stress and heat-related conditions, but also in advancing construction workers’ safety, health, well-being, and QOL. Therefore, the provision of waterlogs by construction and agricultural employers to workers during summer is highly recommended because the regular monitoring of waterlogs by the employer encourages workers to maintain adequate hydration levels, thereby facilitating safety.

The adjustment of full-time workers’ work shifts during summer promotes safety in hot conditions. However, resuming work by 5:00 a.m. may disrupt workers’ sleeping patterns, potentially posing health risks and compromising safety. A qualitative study involving agricultural workers highlighted the vital importance of adequate sleep and emphasized its ability to bolster workers’ resilience against heat stress [29]. In our study, participants worked a 10 h shift for 13 consecutive days, totaling over 60 h a week, which was a significant barrier to safety. The need to reduce both shift duration and the number of working days is crucial to promoting safety and mitigating heat-related risks. Al-Bouwarthan et al. (2020) [28] discovered that working a 10 h shift (60 h/week) increased the incidence of albuminuria among Saudi Arabian residential construction workers compared to workers on a 7 h shift (42 h/week). This finding aligns with Hussen et al.’s (2020) study, which revealed that working over 48 h/week on construction sites elevated dam construction workers’ occupational injury risk by 2.4 times (95% CI (1.55, 3.73)) [33]. Additionally, a systematic review found that long shifts (>8 h shifts) and weekly working hours surpassing 55 h could disrupt circadian rhythms, heighten fatigue level experiences, and severely impair workers’ cognitive efficiency [34]. Such effects can have both short- and long-term repercussions on workers’ safety, health, and overall well-being. Therefore, reassessing workers’ schedules to prioritize safety and minimize the risk of heat stress is strongly recommended.

Generational differences in work habits also created safety concerns, with younger workers perceiving older workers’ extended work periods without breaks as a barrier to safety. These differences may stem from the older workers’ extensive experience and acclimatization to various weather conditions. Our finding is similar to a mixed method study in El Salvador, which found that older sugarcane cutters sometimes neglect rest break instructions [27]. However, given the rising temperatures and increased heat-related risks, educating older workers on the importance of breaks is crucial. Additionally, implementing at least a mandatory break is recommended to ensure all workers, including those on scaffolds, take breaks and the younger generation feels relieved working with the older generation.

Working on the scaffold was the primary barrier to most workers, with fogged spoggles caused by heat. This finding supports previous studies that identified fogged safety glasses and working at heights as contributing factors that elevate HRI risk [8,35]. Providing anti-fog wipes for employees to polish their spoggles is recommended to facilitate safety.

Overall, the implemented heat stress intervention was perceived as effective in promoting workers’ safety and preventing heat-related conditions on this construction site. The program includes training, acclimatization, hydration practices, work–rest cycles, cool-down stations with fans strategically placed across the worksite, emergency preparedness, and daily environmental monitoring using multiple tools, including the wet-bulb globe temperature, the OSHA heat stress app, and other heat index tools like the visible on-site heat index flags and displayed digital heat index level readouts. Additionally, an on-site medical officer conducts medical surveillance to ensure employees are not dehydrated. These employer-provided resources exceeded the OSHA and NIOSH recommendations due to the provision of rest break autonomy, additional resources other than water and electrolytes to keep workers hydrated, cooling towels to suppress CBT, and using multiple tools to monitor the daily work environment. While the study did not explicitly discuss the type of heat stress training workers received, participants mentioned that daily safety toolbox talks and weekly safety meetings served as the main channels for educating workers on heat stress prevention.

This research is the first to qualitatively identify the facilitators and barriers to natural gas construction workers’ safety and evaluate the effectiveness of a heat stress prevention program. Unlike previous studies that focused primarily on heat stress among agricultural workers and its impact on health and productivity, this study emphasized the importance of safe work practices in reducing HRIs and fostering a positive safety culture. Due to limited time, additional safety officers and the other stakeholders (human resources personnel) were not interviewed. Future research should include them to gain a more comprehensive understanding of the program and management’s commitment to workers’ safety. The perceived effectiveness of the program was solely based on the interview transcript and participants’ perceptions. To quantitatively evaluate the program, future studies should analyze on-site recorded heat-related conditions. This study did not specifically differentiate the identified factors based on similar exposure groups. It is important to recognize that not all workers face the same heat stress risk level. Therefore, future research should assess the variation in identified factors by similar exposure groups and tailor intervention strategies accordingly.

## 5. Conclusions

The implementation of an effective heat stress prevention program is fundamental for addressing construction workers’ occupational heat exposure and advancing their safety, health, and well-being. Such a program is a two-way dimension that requires active input and involvement from both employees’ and employer’s management to facilitate safety and safe work practices. Every heat stress prevention program/intervention should encompass multiple components, including a hydration log, which is an identified important facilitator for monitoring and auditing workers’ hydration status on the worksite, along with real-time communication strategies to increase workers’ alert heat levels. Although the effectiveness of the hydration log is yet to be determined, future research should assess this to estimate workers’ daily water consumption volume before nationally incorporating the hydration log into the heat stress prevention program for workers. Additionally, since rest breaks are not mandatory, implementing at least one break with continual heat stress education is essential. Educating workers on the importance of rest breaks and the long-term effects of safe work practices will facilitate safety. Future research should also evaluate the type of heat stress education provided to workers and its effectiveness in promoting workers’ safety, health, and well-being.

## Figures and Tables

**Table 1:** Characteristics of study participants (*n* = 21).

		Focus Groups (*n* = 19)			Interviews (*n* = 2)		
Gender	N (%)	Age Group (Years)			Age Group (Years)		
		18–24	25–54	≥55	18–24	25–54	≥55
Male	15 (86.42)	2	10	1	0	2	0
Female	6 (31.57)	0	5	1	0	0	0
**What job entails**							
Work outside under sun	18 (85.71)	2	14	2			
Work inside and outside	3 (14.28)	2	1				

**Table 2:** Frequency of the most common words that were elicited per data collection method.

Words	6 Focus Groups (*n* = Frequency)	2 Interviews (*n* = Frequency)	Total
Stroke (heat stroke)	19	-	19
Shade (shaded area)	28	-	28
Log (waterlog)	30	27	57
Water	200	63	263
Fruits	31	6	37
Popsicles (electrolyte pops)	26	5	31
Orange	22	-	22
Snow cones	32	4	36
Pickles	17	7	24
Break	133	33	166
Station (cooldown station)	40	4	44
Alcohol, energy drink	15	9	24
Foreman/supervisor	14	26	40
Scaffold	31	4	35
Safety	58	31	89
Ground	18	-	18
Sugar	12	2	14
Care	19	-	19
Lunch	32	-	32
Fan	42	-	42
Company	30	9	39
Harness	20	9	39

**Table 3:** Emerged study themes.

Themes	Subthemes
Heat stress intervention context	Services and resources to prevent heat-related conditions (workplace dynamic for summer)
	Heat stress prevention plan and worker safety
	Employee involvement and safety measures
Factors influencing workers’ safety	Individual work practices and self-care
	Colleagues’ influence on work practices (*co-worker and supervisor*)
	Workplace challenges
Intervention outcome and worker well-being	Intervention influence on worker well-being and QOL
	Yielded positive input

## Data Availability

The data presented in this study are available on request from the corresponding author due to privacy.

## Supplementary Materials

The following supporting information can be downloaded at: https://www.mdpi.com/article/10.3390/ijerph21091255/s1, Table S1: Interview and focus group guide main questions.
