# Supplementary material for: Examining Management and Employees’ Perceptions of Occupational Heat Exposure and the Effectiveness of a Heat Stress Prevention Intervention on Safety and Well-Being among Natural Gas Construction Workers: A Qualitative Field-Based Study
Source: Int J Environ Res Public Health. 2024 Sep 21;21(9):1255. doi: 10.3390/ijerph21091255 (PMC11432089; doi:10.3390/ijerph21091255)
Supplement: Supplementary file 1 [file ijerph-21-01255-s001.zip › ijerph-3185376-supplementary.pdf]

Table S1: Interview and focus group guide main questions.

Interview Guide	Focus Group Guide
1. Can you tell me about the company's heat stress program? (*Knowledge*)<br>2. How do you think the heat stress program influences the workers' safety when working outside? (*Knowledge*)<br>3. If a worker is working from 5:00 a.m. to 3:00 p.m., what can they do to avoid heat-related injuries and illnesses? (*Knowledge*)<br>4. What are some issues workers experience when working under high temperatures outside? *(Experience)*<br>5. How do you think the heat stress program impacts the workers' well-being if they get injured or sick due to exposure to extremely high temperatures while working? *(opinion)*<br>6. How do you know if the program is effective? *(opinion)*	1. What are the things you do to ensure you perform your job safely in a hot environment? *(knowledge)*<br>2. What are the things the company is doing to help you perform your job safely in a hot environment? *(knowledge)*<br>3. When working in a hot environment, what are the things you think make it difficult/easy for you to do your job task safely? *(experience)*<br>4. Can you tell me how you feel working in a hot environment with these factors (i.e., the things that make it easy/difficult for you to do your job safely? *(feeling)*<br>5. How do you think these things (i.e., the above-stated factors) will impact your health if you do/don't work safely in a hot environment? *(opinion)*
